# Effectiveness of health promotion regarding diet and physical activity among Nepalese mothers and their young children: The Heart-health Associated Research, Dissemination, and Intervention in the Community (HARDIC) trial

**DOI:** 10.1080/16549716.2019.1670033

**Published:** 2019-10-01

**Authors:** Natalia Oli, Abhinav Vaidya, Gabriele Eiben, Alexandra Krettek

**Affiliations:** a Department of Internal Medicine and Clinical Nutrition, Institute of Medicine, Sahlgrenska Academy at University of Gothenburg, Gothenburg, Sweden; b Department of Community Medicine, Kathmandu Medical College, Kathmandu, Nepal; c Department of Biomedicine and Public Health, School of Health Sciences, University of Skövde, Skövde, Sweden; d Department of Community Medicine, Faculty of Health Sciences, UiT The Arctic University of Norway, Tromsø, Norway

**Keywords:** Diet, health promotion, mothers, physical activity, young children

## Abstract

**Background**: Nepal, like many low- and middle-income countries, exhibits rising burden of cardiovascular diseases. Misconceptions, poor behavior, and a high prevalence of risk factors contribute to this development. Health promotion efforts along with primary prevention strategies, including risk factor reduction in both adults and children, are therefore critical.

**Objectives**: This study assessed the effectiveness of a health promotion intervention on mothers’ knowledge, attitude and practice (KAP) and their children’s behavior regarding diet and physical activity.

**Methods**: The Heart-health Associated Research, Dissemination and Intervention in the Community (HARDIC), a community-based trial, used peer education to target mothers with 1–9-year-old children in the peri-urban Jhaukhel–Duwakot Health Demographic Surveillance Site, Nepal, during August–November 2016. In the intervention area, 47 peer mothers were trained to conduct four education classes for about 10 fellow mothers (N = 391). After 3 months, all eligible mothers in the intervention and control areas were interviewed and the results were compared with the KAP of all eligible mothers at baseline.

**Results**: Post-intervention, mothers’ KAP median scores had improved regarding heart-healthy diet and physical activity. More mothers had ‘good’ KAP (>75% of maximum possible scores), and mothers with ‘good’ knowledge increased from 50% to 81%. Corresponding control values increased only from 58% to 63%. Mothers’ attitude and practice improved. Additionally, mothers in the intervention area reported improvement in their children’s diet and physical activity behavior. Moreover, Difference in Differences analysis showed that the HARDIC intervention significantly increased mothers’ KAP scores and children’s behavior scores in the intervention area compared to the control area.

**Conclusions**: Our intervention improves KAP scores regarding diet and physical activity and shows potential for expansion via community health workers, volunteers, and/or local women. Moreover, HARDIC can contribute to Nepal’s Package of Essential Noncommunicable Diseases Initiative, which currently lacks a specific package for health promotion.

## Background

Globalization and urbanization contribute to rising global burden of cardiovascular diseases (CVD) which are responsible for one-quarter of deaths worldwide [1,2]. The main health challenge of globalization is changes in health-related behaviors such as diet and physical activity [3,4]. Thus, inadequate fruit and vegetable intake contribute to 2.7 million deaths per year. Hence, 11% of ischemic heart disease occurs due to low fruit and vegetable consumption [5]. Moreover, physical inactivity is responsible for 6–10% of major noncommunicable diseases (NCD) [6]. Every year, 1.9 million people die as a result of physical inactivity [5]. Therefore, dietary and physical activity modification are an important cornerstone of CVD prevention [5]. For instance, reducing salt intake to 6 g/day could annually prevent about 2.5 million deaths globally [6]. Also, a systematic review and meta-analysis shows that an increase in physical activity to recommended WHO levels (at least 150 min of moderate-intensity activity per week) lowers CVD incidence by 17% and the risk of CVD mortality by 23% [7].

In Nepal, an increasingly sedentary behavior and transition from a traditional diet (i.e. high fiber, vegetables, low fat) to a western high-energy dense diet contribute to the rising prevalence of CVD [8]. Experiences from high-income countries (HICs) show that well-planned, community-based health promotion programs can improve lifestyle and avoid premature deaths from CVD among middle-aged people [9,10]. Therefore, the Government of Nepal now prioritizes health promotion for NCDs [11]. The Multi-Sectoral Action Plan for the Prevention and Control of NCDs (2014–2020) emphasizes health promotion and early detection of disease [12]. In 2016, Nepal initiated the Package of Essential Noncommunicable Diseases (PEN) as a pilot program [13]. Compared to strategies that focus only on high-risk people, health promotion activities that target a heart-healthy lifestyle for the entire population potentially have a greater impact on public health [10]. For example, a community-based lifestyle and health promotion intervention that targeted hypertension in a Western Nepal municipality reduced blood pressure in hypertensive individuals [14]. Currently, Nepal’s health research sector lacks studies that explore cardiovascular health knowledge, attitude, and practice (KAP) in the communities, which is an essential starting point for cardiovascular health promotion. The majority of the ongoing studies in Nepal (e.g. the WHO STEPs survey) focus on the manifestation of CVD or their risk factors, while barriers and facilitators for healthy lifestyle remain underexplored [8,15,16].

Our earlier studies in the Jhaukhel–Duwakot Health Demographic Surveillance Site (JD-HDSS) in the Bhaktapur district near Kathmandu, Nepal’s capital city [17], showed a high prevalence of CVD risk factors, including unhealthy diet and low physical activity [18,19]. JD-HDSS also showed a high prevalence of prehypertension (31.8%) in mothers of young children [20]. Further, our qualitative study among mothers in JD-HDSS revealed gaps and misconceptions regarding heart-healthy diet and physical activity [21]. Thus, misunderstandings about the composition of healthy food and an underestimated understanding of the importance of physical activity for health were common. Therefore, health promotional activities need a greater focus on diet and physical inactivity, which are important risk factors for CVD. In Nepal, mothers bear most of the responsibility for their family’s food environment and shape their children’s lifestyle [22]. Preventing risk factors in early childhood is beneficial because habits formed in early life continue into adulthood [23].

Therefore, this study aimed to assess the effectiveness of a health promotion intervention on mothers’ KAP and their children’s behavior as perceived by mothers regarding diet and physical activity.

## Methods

### Study design and participants

The Heart-health Associated Research, Dissemination and Intervention in the Community (HARDIC) community-based trial was designed as a health education on diet and physical activity to promote cardiovascular health in JD-HDSS [17,24]. Participants included mothers with children aged 1–7 years (baseline survey) and 1–9 years (intervention and follow-up). Because a major earthquake, which hit Nepal in April 2015, forced us to delay implementation of the intervention, the gap between baseline (August–November 2014) and intervention (August–November 2016) encompassed 2 years. Compared to baseline, more mothers participated in the follow-up survey, mainly due to an extension of the children’s age range to include the mothers who participated at baseline. Also, the earthquake increased the number of people who moved into the study area [25]. The follow-up study excluded mothers with hearing or mental disorders as well as those whose children were mentally ill or had health conditions that required a special diet and/or physical regimen. Additionally, mothers who had lived in the community for only 2–3 months to perform seasonal work (e.g. in brick kilns) were excluded. Local female community health volunteers assisted in the recruitment of study participants.

### Intervention

**Figure 1.**
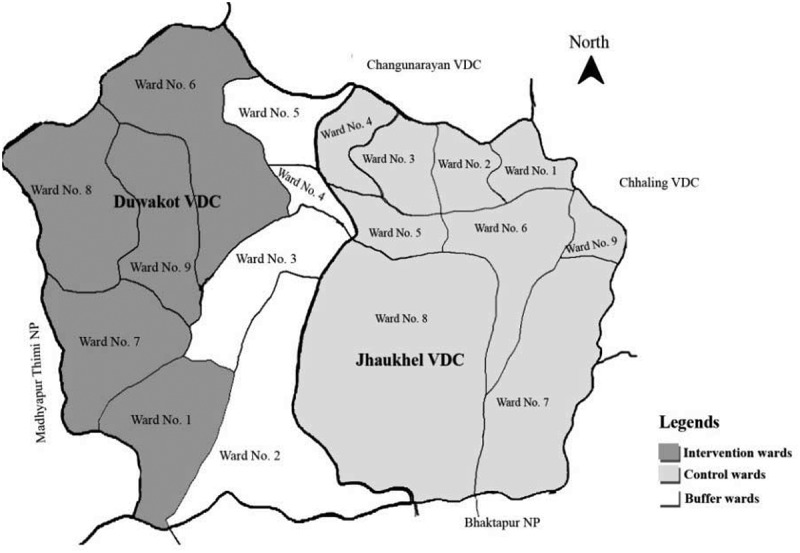
Map of Jhaukhel–Duwakot Health Demographic Surveillance Site showing the intervention area (wards 1, 6, 7, 8, and 9 in Duwakot), control area (all wards in Jhaukhel), and a buffer zone (wards 2, 3, 4, and 5 in Duwakot); VDC, village development committee (according to old federal structure, before 2017–2018).

A lottery method randomly selected Duwakot as the intervention area and Jhaukhel as the control area. All villages in Nepal have nine administrative clusters (wards) [26]. Duwakot and Jhaukhel are neighbouring villages in JD-HDSS. To minimize contamination bias, the five wards selected in Duwakot (intervention) did not border Jhaukhel(control). The control area included all nine wards in Jhaukhel (Figure 1).

The initial plan called for the recruitment of 40 peer mothers (PM) from 440 eligible mothers in the five intervention wards in Duwakot [27]. The PMs would then reach out to 400 fellow mothers (FMs) at a ratio of 1:10. After considering the possibility of dropouts, 47 PMs were recruited among local mothers who volunteered to participate in the program, were available during the entire intervention phase, and had good communication skills. The health promotion program recruited 391 FMs [28]. Importantly, PMs were chosen from different places in all five intervention wards to ensure coverage of all eligible FMs. We interviewed and recruited PMs with the support of local female community health volunteers.

The intervention was based on the findings of the qualitative study with mothers as well as the baseline findings in JD-HDSS, which showed widespread misconceptions about healthy food and the importance of physical activity [21,27]. After reviewing national and international guidelines regarding diet and physical activity and consulting with our international collaborators, finally, the health education intervention program was tailored to the local Nepali context [29,30].

The intervention was based on a combination of health behavior theories that focus on individuals and the community. Health education training was based on the Health Belief Model (HBM), the most commonly used theory to explain health behavior through understanding individuals’ beliefs about health, and Social Cognitive Theory (SCT), which explains the interaction between individuals and their environment [31]. For example, during the training mothers discussed perceived barriers of increasing physical activity and possible solutions to reduce such barriers. The mothers’ intentions were considered an important output of the intervention. The Diffusion of Innovations Theory was useful because it communicates knowledge and skills obtained via training and educational classes to other members of the community [31].

The HARDIC health education intervention covered basic aspects of heart disease and focused on diet and physical activity. The intervention package consisted of seven modules (basic concept of cardiovascular health; fruits and vegetables; fibers; fats; salt; sugar and soft drinks; obesity and physical activity), which were published as a training manual, and a set of flip charts and posters. All educational material was developed in English and then translated into Nepali language.

During the HARDIC intervention, mothers received health education to improve their KAP for diet and physical activity. The intervention emphasized improved self-efficacy by addressing existing barriers to a healthy lifestyle. To empower and motivate the locally selected and trained PMs who would educate neighboring mothers in the community, participation was defined as a component of health promotion. Thus, the intervention applied peer education at two levels: (i) team selection and training of local PMs, and (ii) health education classes given by PMs in their own households to FMs in their own neighborhood.

The intervention comprised two rounds for PMs and FMs during August–November 2016 and included a one-month gap between rounds. The research team trained PMs (four hours/day for six consecutive days) using interactive lectures, practical group work, demonstrations, and assignments, as described earlier [28]. During Round 1, trained PMs conducted four educational classes for groups of approximately 10 FMs. In Round 2, PMs conducted one class under the supervision of four field supervisors, who assisted and monitored all field activities. Simultaneously, all steps of the intervention underwent a process evaluation that included the immediate impact of HARDIC on mothers’ knowledge [28].

### Follow-up

**Figure 2.**
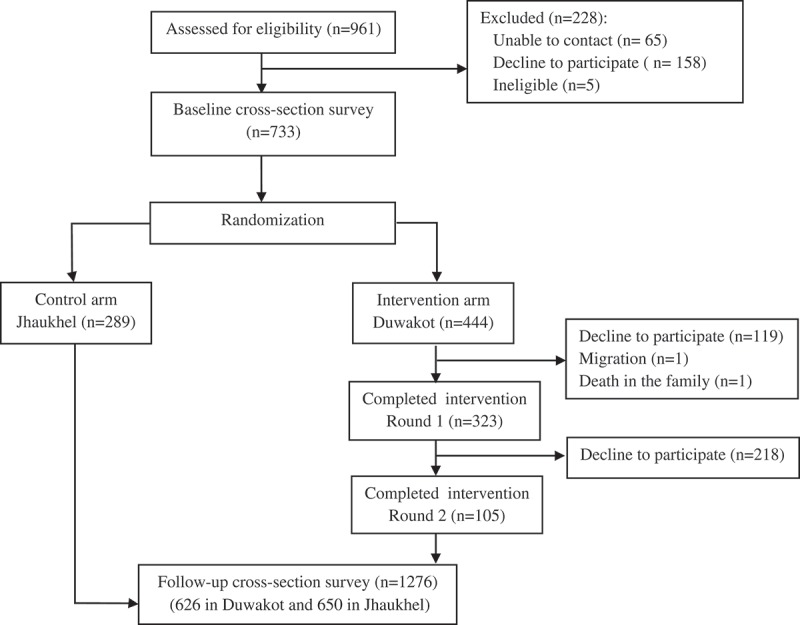
Enrollment, intervention allocation and follow-up of the mothers.

Using the same questionnaire that was developed and applied for the baseline study and described previously [27], the follow-up survey measured the effect of the intervention on mothers’ KAP for diet and physical activity. It also assessed the possible impact of the intervention on children’s behavior regarding diet and physical activity, based on the perception of their mothers. Aided by the main supervisor and three field supervisors, the first author (NO) recruited and trained nine enumerators. The enumerators collected data during January–February 2017 via door–to-door visits and interviews with all eligible mothers. A lottery selected one respondent from households containing more than one eligible mother. Mothers who were absent during the household visit were contacted by phone and interviewed later. Enrollment, intervention allocation and follow-up of the mothers are shown in Figure 2.

### Ethical considerations

Ethical approval was given by the Nepal Health Research Council (No. 150/2014). This trial is registered with ClinicalTrials.gov (No.NCT03639402). The trained enumerators obtained informed consent from each respondent. All interviewees were informed that they could leave the interview at any point. Confidentiality was strictly maintained. Each respondents received 300 Nepalese rupees (NPR) (1 USD = 101.6 NPR) as an incentive to participate in the follow-up survey. During follow-up, all eligible mothers in the control area received government-published pamphlets on NCDs and behavioral risk factors.

### Statistical analysis

Data entry and analysis were conducted using the Statistical Package for the Social Sciences (SPSS), version 23.0 (IBM, Armonk, New York, USA). In families with more than one child aged 1–7 years, the oldest child was selected for data analysis regarding behavior. We scored KAP responses as previously described [27] and combined relevant scores to calculate composite scores for mothers’ KAP as well as for children’s behavior. Mothers’ KAP scores and children’s behavior scores were divided into three categories based on a percentage of maximum possible scores: ‘good’ (76–100%), ‘fair’ (51–75%), and ‘poor’ (0–50%). The maximum possible scores for mothers’ KAP were 73, 192, and 17, respectively, and the maximum score for children’s behavior was 110 [27]. Due to non-normal data distribution, median and interquartile range (IQR) for KAP scores were calculated for diet and physical activity. The Mann–Whitney U test compared baseline and follow-up results from Duwakot and Jhaukhel. A Chi-squared test compared mothers’ sociodemographic variables in the intervention and control areas at baseline and at follow-up. *P* < 0.05 was considered statistically significant.

Changes in diet and physical activity were analyzed post-intervention for all eligible mothers in JD-HDSS. The mothers control group was used to adjust for background variables and other unmeasured confounders that could affect KAP. Due to similarities in the health systems of Jhaukhel and Duwakot, both villages received nearly identical health-related information in the absence of intervention. Thus, the Difference in Differences (DiD) model was used to estimate changes between the villages [32,33].

Outcomes at follow-up and baseline were compared between the intervention and control areas. The effect of an intervention on outcomes can be estimated from the difference at baseline and follow-up in the intervention area and the difference at baseline and follow-up in the control area. Subtraction of those differences in outcomes yields the ‘difference in differences,’ which identifies the real effect of the intervention [32]. DiD estimates were obtained from a linear regression model [34], which was also used to explore the association of the impact of the intervention with various demographic variables of mothers (age, education and occupation) and children (age and sex).

## Results

Eligible mothers at baseline (N = 733) included 444 from five intervention wards in Duwakot and 289 in the control area (all wards of Jhaukhel). The cross-sectional follow-up survey covered all eligible mothers irrespective of participation in the intervention. During follow-up, the number of eligible mothers had increased to 1276 (626 and 650 in Duwakot and Jhaukhel, respectively) due to the extended age range of eligible mothers and increased migration to the study area following the major earthquake in April 2015 (Table 1).

**Table 1.** Sociodemographic characteristics of mothers in intervention (Duwakot) and control (Jhaukhel) areas during baseline and follow-up.

	Baseline study (*N* = 733)			Follow-up study (N = 1276)		
Mothers’ variables	Duwakot *N* (%)	Jhaukhel *N* (%)	*P* value^a^	Duwakot *N* (%)	Jhaukhel *N* (%)	*P* value${\;}^{*}$
Age (years)						
≤25	104 (23.4)	62 (21.5)	0.801	123 (19.6)	130 (20.0)	0.752
26–35	305 (68.7)	205 (70.9)		409 (65.3)	432 (66.5)	
≥36	35 (7.9)	22 (7.6)		94 (15.0)	88 (13.5)	
Education						
<Grade 5	120 (27.0)	56 (19.4)	0.004	137 (21.9)	124 (19.1)	0.376
Grade 5–10	218 (49.1)	135 (46.7)		301 (48.1)	334 (51.4)	
>Grade 10	106 (23.9)	98 (33.9)		188 (30.0)	192 (29.5)	
Religion						
Hindu	405 (91.2)	268(92.7)	0.370	582 (93.0)	607 (93.4)	0.833
Buddhist	25 (5.6)	10 (3.5)		30 (4.8)	27 (4.2)	
Others^b^	14 (3.2)	11 (3.8)		14 (2.2)	16 (2.5)	
Ethnicity^c^						
Newar	115 (25.9)	115 (39.8)	< 0.001	142 (22.7)	305 (46.9)	< 0.001
Brahmin	66 (14.9)	86 (29.8)		76 (12.1)	117 (18.0)	
Chhetri	169 (38.1)	41(14.2)		273 (43.6)	103 (15.8)	
Other major hill castes^d^	72 (16.2)	42 (14.5)		121(19.3)	115 (17.7)	
Others^e^	22 (5.0)	5 (1.7)		14 (2.2)	10 (1.5)	
Family structure						
Nuclear	275 (61.9)	121(41.9)	< 0.001	542 (86.6)	456 (70.2)	< 0.001
Extended	169 (38.1)	168 (58.1)		84 (13.4)	194 (29.8)	
Mothers’ main occupation						
Agriculture	21(4.7)	3 (1.0)	0.009	29 (4.6)	99 (15.2)	< 0.001
Office	15 (3.4)	9 (3.1)		23 (3.7)	19 (2.9)	
Labor	37 (8.3)	15 (5.2)		37 (5.9)	45 (6.9)	
Self-employed	48 (10.8)	46 (15.9)		61 (9.7)	96 (14.8)	
Housewife	323 (72.7)	216 (74.7)		476 (76.0)	391(60.2)	
Average monthly household income (NPR)^f^						
< 10,000	92 (22.2)	88 (32.7)	0.015	74 (11.9)	137 (21.2)	< 0.001
10,000–19,999	209 (50.4)	113 (42.0)		330 (53.0)	353 (54.7)	
20,000–29,999	56 (13.5)	42 (15.6)		107 (17.2)	105 (16.3)	
30,000–39,999	27 (6.5)	11 (4.1)		46 (7.4)	16 (2.5)	
> 40,000	31 (7.5)	15 (5.6)		66 (10.6)	34 (5.3)	
Total	**444**	**289**		**626**	**650**	

^a^Obtained from a χ^2^ test

^b^Other religion includes Christianity and Islam.

^c^Classification of ethnic groups is based on the National Central Bureau of Statistics in Nepal [35].

^d^Other major hill castes include Tamang, Dalit, Thakuri, Magar, and Rai.

^e^Other ethnicity includes Lama, Sherpa, Madeshi, Gurung, Tharu.

^f^NPR = Nepalese rupees (1 USD = NPR 101.6 approximately).

### Sociodemographic characteristics

Table 1 shows the sociodemographic characteristics of the mothers at baseline and follow-up and also compares the intervention and control areas. Although ethnicity, family structure, mothers’ occupation, and household income differed significantly at baseline and follow-up, observed differences for education occurred only at baseline.

Mothers’ median (IQR) age was 28 (5) years and 30 (7) years at baseline and follow-up, respectively (*P* < 0.001). Age range was 19–48 years at baseline and 18–48 years at follow-up. Children’s median (IQR) age was 3 (3) and 5 (4) years at baseline and follow-up, respectively (*P* < 0.001). The percentage of girls at baseline did not differ significantly from that at follow-up (45.3% vs.44.9%, respectively).

### The effectiveness of a health promotion intervention on diet and physical activity among mothers and their children

#### Mothers’ KAP categories

Mothers’ KAP scores were assessed before and after the intervention as categories and also as continuous variables. The intervention improved mothers’ KAP in Duwakot (intervention area). Mothers with ‘good’ knowledge in Duwakot increased 31% (from 50% at baseline to 81%) at follow-up compared to Jhaukhel (control), where ‘good’ knowledge increased 5% (from 58% to 63%).

Similarly, the percentage of mothers with ‘good’ attitude increased 20% (from 43% to 63%) in Duwakot, but remained almost the same in Jhaukhel at baseline and follow-up (45% and 48%, respectively). Additionally, the intervention improved mother’s practice. At baseline, neither village had mothers with ‘good’ practice. Post-intervention, 9% of mothers in Duwakot showed ‘good’ practice compared to 0% in Jhaukhel.

#### Mothers’ KAP scores

**Figure 3.**
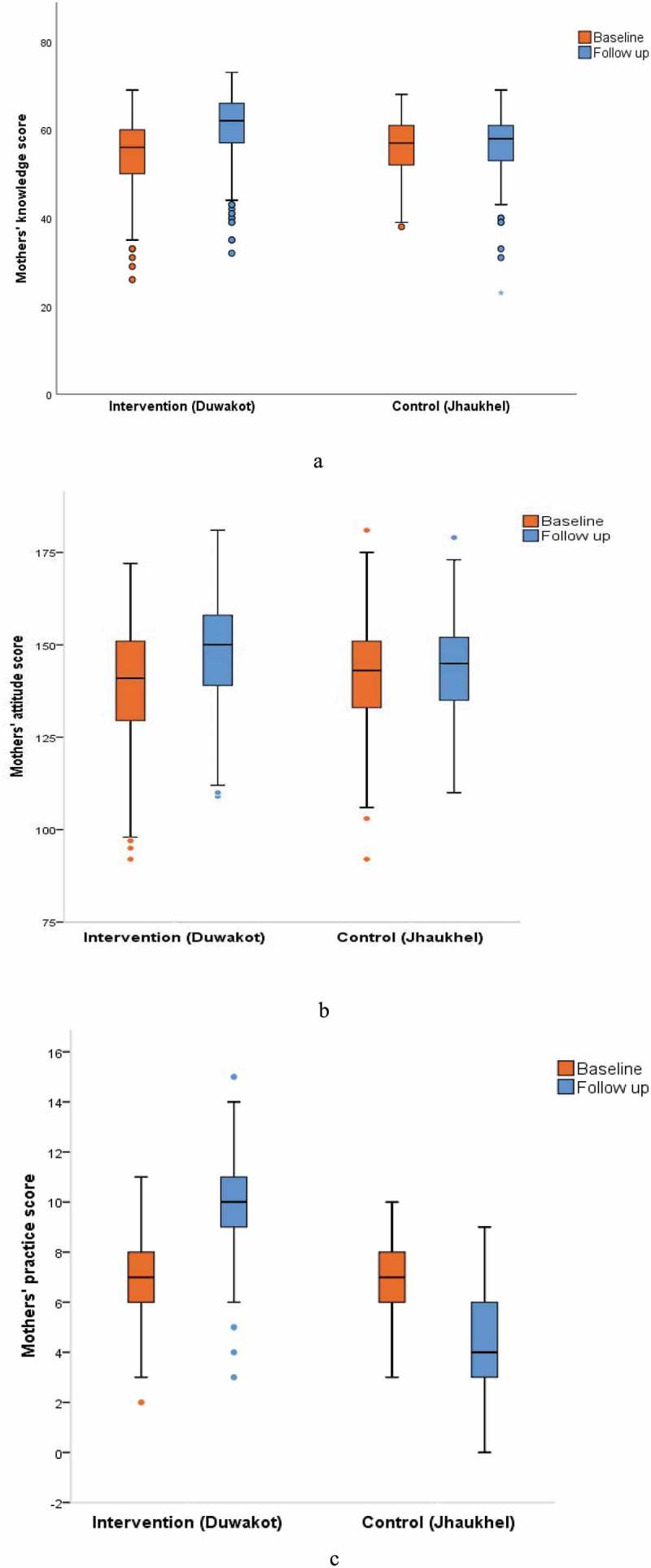
Effectiveness of the health promotion intervention on mothers’ knowledge (a), attitude (b), and practice (c) regarding diet and physical activity. Note: Maximum possible scores for knowledge, attitude, and practice were 73, 192 and 17, respectively.

KAP scores were analyzed as continuous variables. At baseline, median (IQR) KAP scores were 56 (10), 141 (22), and 7 (2), respectively, in Duwakot compared to 57 (9), 143 (18), and 7 (2), respectively, in Jhaukhel. At follow-up, median (IQR) KAP scores in Duwakot were 62 (9), 150 (19), and 10 (2) compared to 58 (8), 145 (17), and 4 (3) in Jhaukhel. Figure 3 shows the changes in mothers’ KAP scores in the intervention and control areas from baseline to follow-up.

#### Children’s behavior scores

**Figure 4.**
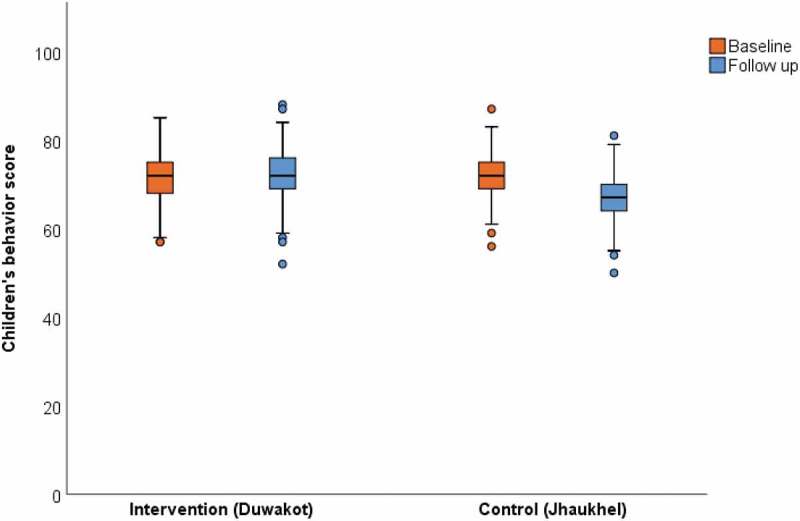
Effectiveness of the health promotion intervention on children’s behavior. Maximum possible score = 110.

To explore the effectiveness of the intervention on children, median (IQR) behavior scores for diet and physical activity were compared in the intervention and control areas at baseline and follow-up (Figure 4). The median (IQR) score was the same (72 (7)) in the intervention area at baseline and follow-up. In the control area, the median (IQR) behavior score was 72 (6) at baseline and 67 (6) at follow-up.

### DiD analysis of the health promotion intervention

Table 2 shows the effectiveness of the intervention as calculated using DiD estimates from a linear regression model. In contrast to the control area, mothers in the intervention area significantly improved their KAP scores post-intervention. DiD estimates also showed that children’s behavior regarding diet and physical activity improved in the intervention area.

**Table 2.** Effectiveness of the intervention on mothers’ KAP and children's behavior as perceived by mothers regarding diet and physical activity.

	Intervention area (Duwakot)		Control area (Jhaukhel)		Impact	
	Baseline mean (SD)	Follow-up mean (SD)	Baseline mean (SD)	Follow-up mean (SD)	Difference in differences	95% CI
Knowledge	54.6 (7.5)	61.2 (6.7)	56.2 (6.2)	56.9 (6.0)	5.8	(4.6; 7.0)
Attitude	139.8 (15.3)	148.2 (12.7)	141.7 (13.1)	143.3 (11.6)	6.9	(4.5; 9.3)
Practice	6.7 (1.4)	10.1 (1.8)	7.1 (1.3)	4.5 (2)	6.0	(5.6; 6.3)
Children’s behavior	71.6 (4.9)	72.1 (5.2)	71.9 (4.7)	67.1 (4.3)	5.2	(4.3; 6.1)

Effectiveness of the intervention was measured using DiD estimates from a linear regression model.

### Effect of mothers’ and children’s demographic variables on outcome of the health promotion intervention (DiD estimates)

Table 3 compares the effect of the intervention on mothers’ KAP, as determined by subgroup analysis of mother’s demographic variables (i.e. age, education, and occupation). Compared to younger mothers, those aged >35 years showed no improvement in knowledge about diet and physical activity. However, the difference in KAP for knowledge was not significant in mothers aged 26–35 years old and <25 years. We observed no significant difference in KAP for attitude and practice, irrespective of age.

**Table 3.** DiD analysis according to mothers’ and children’s demographic variables.

	Mothers			Children
	Knowledge DiD (95%CI)	Attitude DiD (95%CI)	Practice DiD (95%CI)	Practice DiD (95%CI)
Mothers’ variables				
Age (years)				
≤25	6.0 (3.1; 8.8)	4.4 (−1.1; 9.8)	5.7 (5.0; 6.4)	4.4 (2.6; 6.2)
26–35	6.4 (5.0; 7.8)	8.1 (5.2; 10.9)	6.0 (5.6; 6.4)	5.3 (4.2; 6.4)
≥36	0.2 (−4.1; 4.4)	2.5 (−5.6; 10.6)	6.2 (5.2; 7.2)	6.6 (3.7; 9.4)
Education				
<grade 5	5.2 (2.5; 7.9)	4.1 (−1.0; 9.2)	6.4 (5.8; 7.1)	3.4 (1.6; 5.3)
grade 5–10	4.5 (2.9; 6.2)	4.7 (1.4; 8.0)	5.6 (5.1; 6.0)	5.1 (3.8; 6.3)
>grade 10	5.7 (3.9; 7.5)	6.9 (3.0; 10.8)	6.2 (5.6;6.8)	6.4 (4.8; 8.1)
Main occupation				
Agriculture	4.8 (−3.1; 12.8)	16.7 (1.2; 32.2)	5.1 (2.9; 7.3)	−1.7 (−7.9; 4.5)
Office	−0.7 (−6.4; 5.0)	0.3 (−12.4; 12.9)	6.0 (4.2; 7.9)	5.8 (0.5; 11.1)
Labor	1.8 (−4.1; 7.5)	4.0 (−6.6; 14.6)	5.9 (4.8; 6.9)	2.2 (−1.8; 6.2)
Self-employed	3.5 (0.6; 6.5)	7.2 (1.1; 13.3)	5.3 (4.4; 6.3)	6.9 (4.2; 9.5)
Housewife	6.0 (4.6; 7.4)	4.5 (1.7; 7.3)	6.1 (5.7; 6.5)	5.5 (4.5; 6.5)
Children’s variables				
Age (years)				
1–4	5.7 (4.1; 7.3)	3.8 (0.7; 7.0)	5.6 (5.2; 6.1)	5.6 (4.4; 6.7)
5–9	5.5 (3.5; 7.6)	10.4 (6.4; 14.4)	6.4 (5.9; 6.9)	4.6 (3.1; 6.1)
Sex				
Male	5.1 (3.4; 6.8)	6.3 (3.1; 9.6)	5.9 (5.4; 6.3)	4.7 (3.5; 5.9)
Female	6.7 (5.0; 8.5)	7.4 (3.8; 11.0)	6.1 (5.7; 6.6)	5.8 (4.5; 7.2)

DiD = Difference in Differences.

The effectiveness of the intervention was measured using DiD estimates from a linear regression model. Numbers show DiD of mean score between intervention and control areas at baseline and follow-up across various subcategories of mothers’ and children’s demographic variables.

Analysis of the effectiveness of the HARDIC intervention according to mothers’ education level found no significant differences between levels. Hence, mothers improved their KAP scores irrespective of education. Similar to previous variables, occupation did not significantly affect outcome of the intervention. However, housewives showed the highest scores for knowledge and practice, and self-employed mothers had the highest attitude scores compared to other occupations. Analysis of the effectiveness of the HARDIC intervention on children’s behavior, as perceived by their mothers, determined that children’s behavior was independent of their age and sex and changes in the mothers’ knowledge and practice did not associate with children’s age (Table 3). However, mothers of children aged 5–9 years significantly improved their attitude compared to mothers of younger children. Also, mothers’ KAP scores were independent of sex of the children.

## Discussion

In this paper, we demonstrate that the HARDIC health promotion intervention improved mothers’ KAP regarding diet and physical activity in a peri-urban community of Nepal. Compared to the control village Jhaukhel, KAP scores in the intervention village Duwakot increased significantly from baseline to follow-up. Similar positive changes have been reported in Iran, where the diet of mothers and young children was enhanced by a community-based intervention program [36]. A community-based intervention in China also yielded a positive effect on physical activity as well as fruit and vegetable consumption [37].

Primarily, the HARDIC intervention changed KAP regarding diet and physical activity among mothers with young children. Compared to baseline, when no mothers in either village had ‘good’ practice, 9% showed ‘good’ practice in Duwakot post-intervention vs. no changes for mothers in Jhaukhel. Compared to baseline, lower median (IQR) scores for mother’s practice in the control area at follow-up could be attributed to newly migrated mothers with poorer practice that may have influenced their children’s behavior. Poor dietary and physical activity practice may also be a consequence of the major earthquake that hit Nepal in April 2015. Similarly, the HARDIC intervention likely neutralized a similar trend of poor dietary and physical activity practice in the intervention area.

Second, DiD analysis showed significant improvement in children’s behavior regarding diet and physical activity (DiD = 5.2; 95% CI 4.3; 6.1) as perceived by their mothers. However, the median (IQR) score for children’s behavior (72[7]) was identical in the intervention area at baseline and at follow-up. The significant improvement of children’s behavior in the intervention area is possibly due to a lower median (IQR) behavior score in the control area at follow-up compared to baseline (67[6] vs 72[6], respectively) due to the same reasons as for mothers. DiD analysis also adjusted for possible effects of mother’s and children’s sociodemographic variables (e.g. mothers’ age, education, and occupation and children's age and sex) on the effectiveness of the intervention.

Earlier studies reported that education has a positive association with KAP [27,37]. However, our pre-intervention studies determined many gaps and misconceptions regarding heart-healthy diet and physical activity, even among well-educated mothers, in JD-HDSS [21,27]. The HARDIC intervention was designed to provide all eligible mothers, irrespective of education level, with the basic concept of CVD and its prevention through healthy diet and physical activity. Our DiD analysis showed that the intervention program improved KAP of all mothers equally, irrespective of education level. Moreover, population changes among differential proportions of educated mothers at baseline and follow-up in Duwakot and Jhaukhel did not significantly influence the effect of the intervention, suggesting that the study achieved its goal of developing and providing a health education intervention that reaches all mothers, irrespective of education level.

Additionally, most (about 60%) PMs and FMs were housewives themselves, possibly explaining the higher effectiveness of the HARDIC intervention on the knowledge and practice of housewives [28]. PMs scheduled educational classes for FMs according to the convenience of mothers’ groups, likely making it problematic for working mothers to participate. Further, the intervention affected knowledge and attitude more in mothers aged >35 years compared to younger mothers. The effect of the intervention on mothers’ KAP and children’s behavior was independent of children’s age and sex.

The HARDIC trial was designed using a peer education approach, which has earlier proved efficient in improving dietary knowledge and behavior and promoting better maternal and neonatal care practices in rural areas of Nepal and other countries [14,39,40]. Because the use of behavior theories during the development of a health promotion program improves the likelihood of positive behavior changes [41], the HARDIC intervention was based on health behavior theories that target both individuals (i.e. HBM and SCT) and the community (i.e. the Diffusion of Innovations Theory) [31]. HARDIC targeted mothers’ KAP regarding diet and physical activity at both levels. HBC addresses problem behavior by inducing health self-consciousness and motivating mothers to change their behavior, focusing on understanding an individual’s perceived benefit, barriers towards changes, perceived susceptibility, and perceived severity of the disease. Thus, use of HBM in our study increased understanding of mothers’ perception of diet and physical activity. On the other hand, SCT emphasizes the interaction between human behavior, an individual’s personal factors, and environmental factors by focusing on self-efficacy (i.e. confidence in one’s ability to take action and overcome barriers) [31]. Therefore, we also emphasized SCT during the intervention, as recently described in the HARDIC process evaluation paper [28]. Using a peer mother approach worked as a ‘learning from others’ component of SCT in the HARDIC trial.

The Diffusion of Innovations Theory was applied in the intervention area to disseminate knowledge and skills to mothers of young children. Community participation was an important and effective component, motivating and empowering local PMs to spread the knowledge and skills they received during training to other mothers in the community [42]. Other studies have demonstrated that Diffusion of Innovations Theory successfully disseminates knowledge and skills among mothers [14,43].

### Strengths and limitations

Community-based trials have limitations, especially in low-income countries like Nepal. Tracking is difficult due to the absence of house numbers and street names, and the high rate of internal and international migration causes population instability [34]. Because the 2015 earthquake increased the number of people who moved into the study area to flee destroyed houses, more eligible mothers participated in the follow-up survey compared to baseline (1276 vs. 733, respectively). Additionally, we extended the upper limit of eligible age for children (from 7 years to 9 years) to include all mothers who participated during baseline, 2 years before the intervention [27]. Moreover, maintaining the same lower age limit (1 year) for the children at follow-up allowed for an intervention with the maximum number of mothers who potentially could benefit from HARDIC.

This study has some limitations. First, the trial was limited to only two rounds of intervention. Also, the follow-up study was conducted 3 months after the intervention and therefore was only able to demonstrate short-term impact. An expected long-term outcome of the HARDIC trial would include reduced prevalence of CVD in JD-HDSS. Importantly, sustaining KAP regarding diet and physical activity takes time and requires regular rounds of health promotional intervention.

Second, children’s behavior regarding diet and physical activity was assessed by their mothers’ perception, not directly with the children. Hence, there is a possibility of bias in the interpretation of results.

Third, the study may include contamination bias because the intervention and control areas were in neighboring villages. However, we selected five wards in Duwakot that did not border the control area and remaining wards formed a buffer zone that minimized contamination bias.

Finally, mothers’ sociodemographic differences (i.e. ethnicity, family structure, mothers’ occupation, and household income) in the intervention and control areas were confounders in this study. Although DiD analysis mitigates the effect of confounding factors and selection bias, it has its own limitations. Despite assuming the absence of factors that disproportionately affect intervention or control groups during implementation, some factors may not be taken into account in the regression, especially when the composition of a population changes over time. Hence, there is the potential of bias in estimating the impact of the program.

Nonetheless, our study has several strengths. To our knowledge, it is the first community–based trial in Nepal to address diet and physical activity among mothers with young children. The approach of the trial and active community participation yielded improved confidence and decision-making power among the mothers [28]. This is one aspect of sustainability of the changes due to the intervention. Also, our strategy for recruiting PMs ensured representation in all interventional wards. It also ensured the participation of FMs with low socioeconomic status.

Furthermore, HARDIC can contribute to Nepal’s PEN initiative (2016) [13]. In Nepal, PEN considers health promotion and lifestyle changes, including diet and physical activity, but there is no specific health promotional package for the community level. Moreover, Nepal’s health research sector still needs more community-based scientific evidence regarding cardiovascular health and its risk factors, which are crucial for initiating cardiovascular health promotion in the country.

## Conclusion

The HARDIC health promotion intervention improved mothers’ KAP regarding diet and physical activity. It also has potential for scaling up in other settings. In other settings, training female community health volunteers, paramedics, health assistants, and auxiliary health workers could contribute to Nepal’s PEN initiative. Moreover, collaboration with the education sector (e.g. community schools) in health promotion interventions could further improve children’s behavior by targeting factors that affect their lifestyle outside the family environment.

## Paper context

High prevalence of risk factors contributes to the rising burden of cardiovascular diseases in Nepal. Nepal currently lacks community interventions on diet and physical activity, and does not prioritize prevention of cardiovascular diseases from a young age. The HARDIC health promotion intervention, which is based on the peer education approach, improved mothers’ KAP for diet and physical activity. The intervention has potential for use in other sub-populations and low-resource settings.
